# Post-appendectomy Adhesive Small Intestine Obstruction With Gangrene: A Sinister Case

**DOI:** 10.7759/cureus.39437

**Published:** 2023-05-24

**Authors:** Siddharth Sankar Das, Suhasini Krishnan, Moaza Hashim Albedwawi, Walid Bondok, Husni Shalak

**Affiliations:** 1 General Surgery, Dubai Hospital, Dubai, ARE; 2 Medicine, Dubai Academic Health Corporation, Dubai, ARE

**Keywords:** band, ischemia, small intestine, gangrene, obstruction, adhesions

## Abstract

Intestinal adhesions are fibrotic bands of scar tissue that develop intra-abdominally due to serosal or peritoneal irritation caused during surgery or by severe infections. It may also occur congenitally. It can lead to serious complications such as small bowel obstruction, which is then termed adhesive small bowel obstruction. In this scenario, it can constrict the bowel wall and cause ischemia and necrosis of the affected intestinal segment. Computed tomography imaging may show characteristic signs, such as the “whirl sign” or “fat-bridging sign.” Diagnostic laparoscopy or laparotomy can confirm the diagnosis and presence of adhesions. Management of this condition is either conservative or surgical, the latter of which is necessary in the case of intestinal strangulation. While the literature supports the laparoscopic method of adhesiolysis, practically, it may present technical difficulties. Surgeons should employ their clinical judgment in cases where an open procedure may be more beneficial. We present a case of this very occurrence and discuss the risk factors, pathogenesis, diagnostic evaluation, and, finally, the approaches to surgical management of this condition.

## Introduction

Intestinal adhesions are fibrotic bands of scar tissue that unusually connect two or more intra-abdominal organs. This scar tissue can form due to tissue irritation or disturbances, such as surgery, significant trauma, or any infectious disease. Although it is a normal phenomenon in the healing phase, it can pose a significant risk in the development of bowel obstruction [1]. Postoperative intra-abdominal adhesions account for nearly half of all cases of acute intestinal obstruction, with nearly 70% involving the small intestine [2]. The following types of surgeries carry a 63% to 97% risk of forming intestinal adhesions: appendectomies, colonic procedures, and gynecological surgeries [2,3]. Adhesive small bowel obstruction (ASBO) causes approximately 16% of surgical admissions and significantly increases morbidity and mortality due to its high risk of recurrence. These adhesion bands can constrict the bowel wall, leading to ischemia and necrosis of the affected segment [3]. Described below is a case of this very phenomenon.

## Case presentation

A 35-year-old female patient presented to the emergency department with complaints of sudden, severe, and diffuse abdominal pain for one day, associated with more than twenty episodes of vomiting. The patient denied any changes in bowel movements, such as constipation and obstipation, any fever or chills, or abdominal distension. The patient denied any past history of abdominal pain or any previous hospital admission. Her past surgical history includes an open appendectomy performed 25 years ago. Laboratory investigations were unremarkable. The urine pregnancy test reported negative results. Upon physical examination, the patient looked dehydrated, with diffuse abdominal tenderness on palpation and sluggish bowel sounds on auscultation.

**Figure 1:**
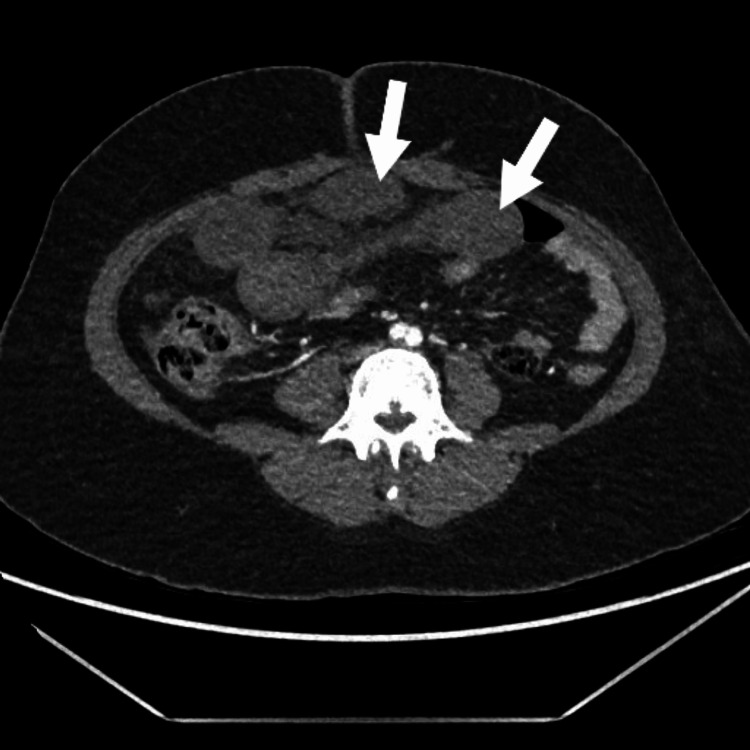
Circumferential wall thickening in multiple segments of the distal small bowel (axial view).

**Figure 2:**
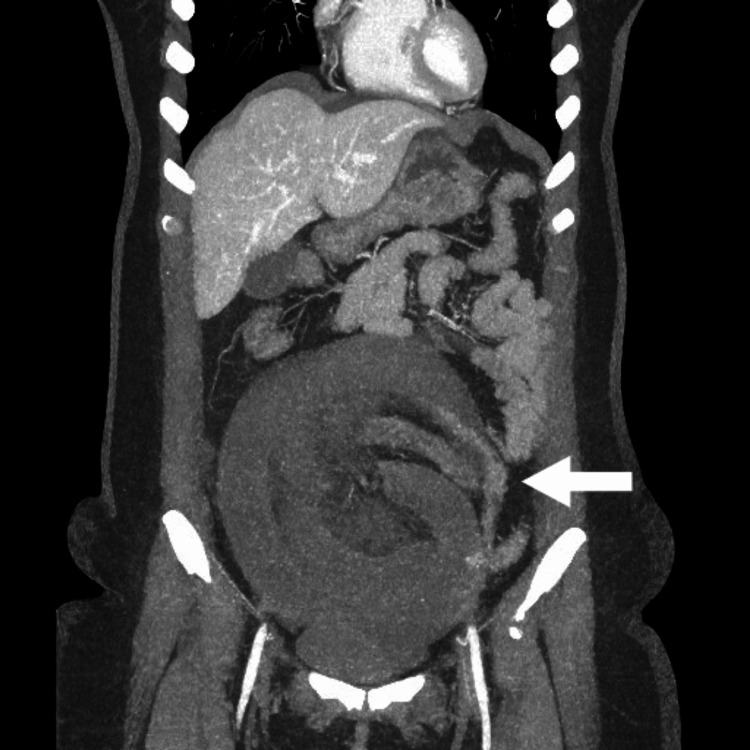
Twisted and dilated intestinal loops with non-enhancement of the affected segment (coronal view).

An abdominal computed tomography (CT) angiogram revealed long-segment circumferential wall thickening in multiple segments of the distal small bowel (distal jejunal and proximal ileal loops, central and lower abdomen) and poor enhancement of these thickened bowel loops with a lack of differentiation of different layers of the bowel wall despite an adequate bolus of intravenous contrast, as seen in Figure 1. There is also dilatation and twisting of the intestinal loops with no enhancement of the wall of the affected segment, as seen in Figure 2. While the superior mesenteric artery is patent from the origin until it gives terminal branches, the vasa recta is not well opacified. There is also an increase in free fluid in the abdomen and pelvis. The imaging features, in conjunction with the history, strongly suggest small bowel obstruction and gangrene of the affected loops of the intestine.

**Figure 3:**
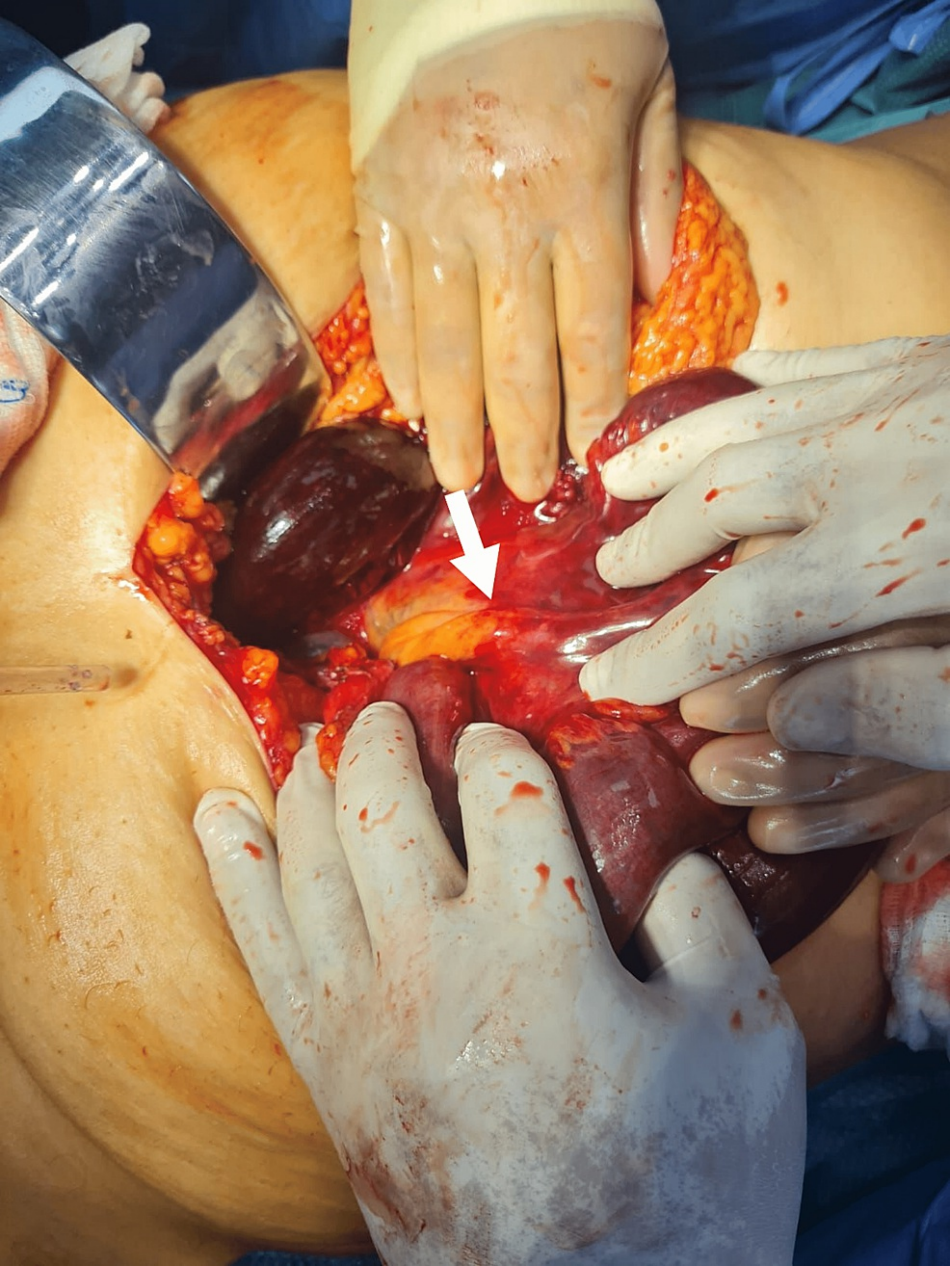
Enlargement of the adhesion band causing twisting of intestinal loops.

**Figure 4:**
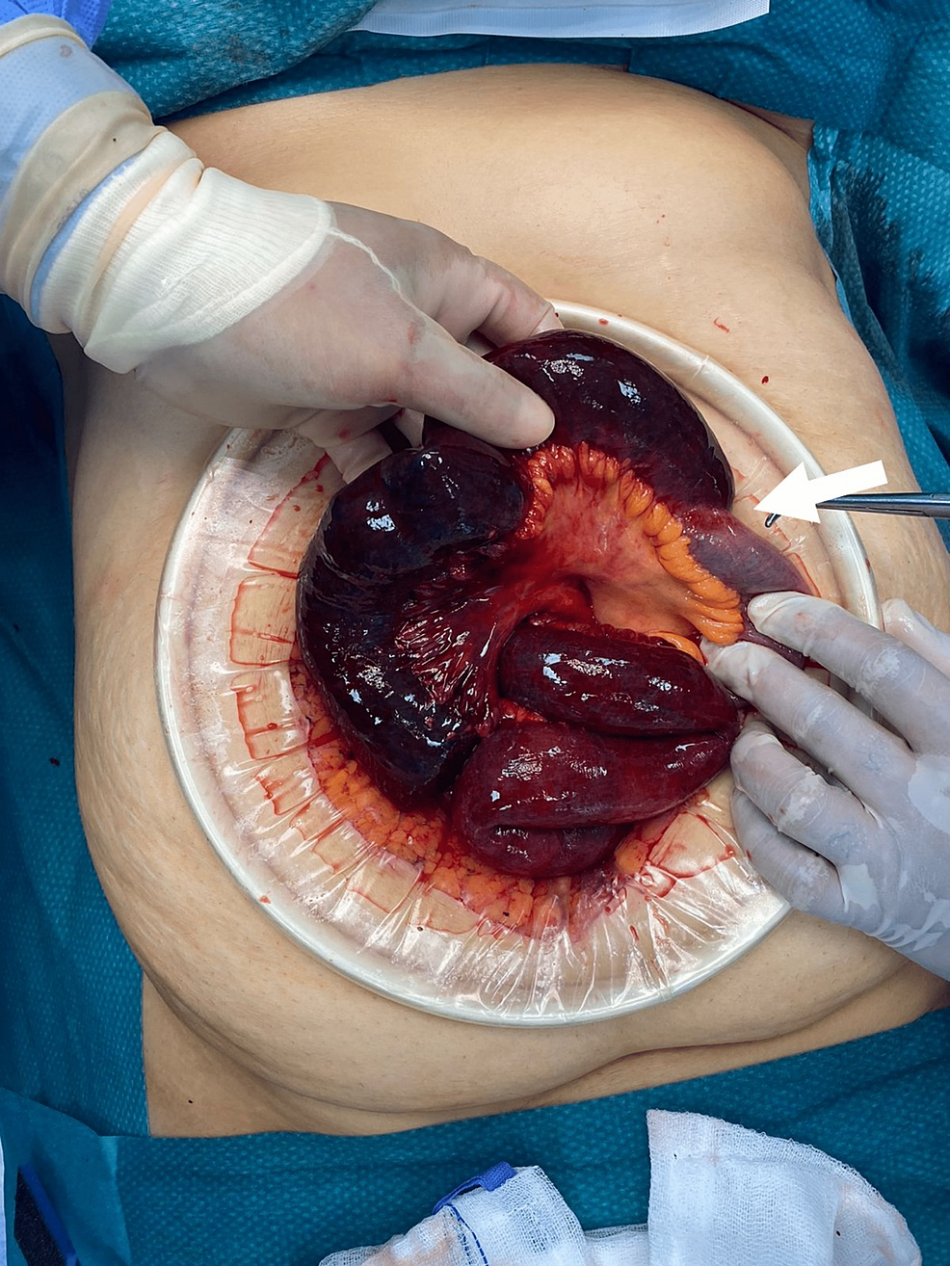
Post band release and detorsion of intestines showing compression effect resulting in gangrene of intestinal segment.

**Figure 5:**
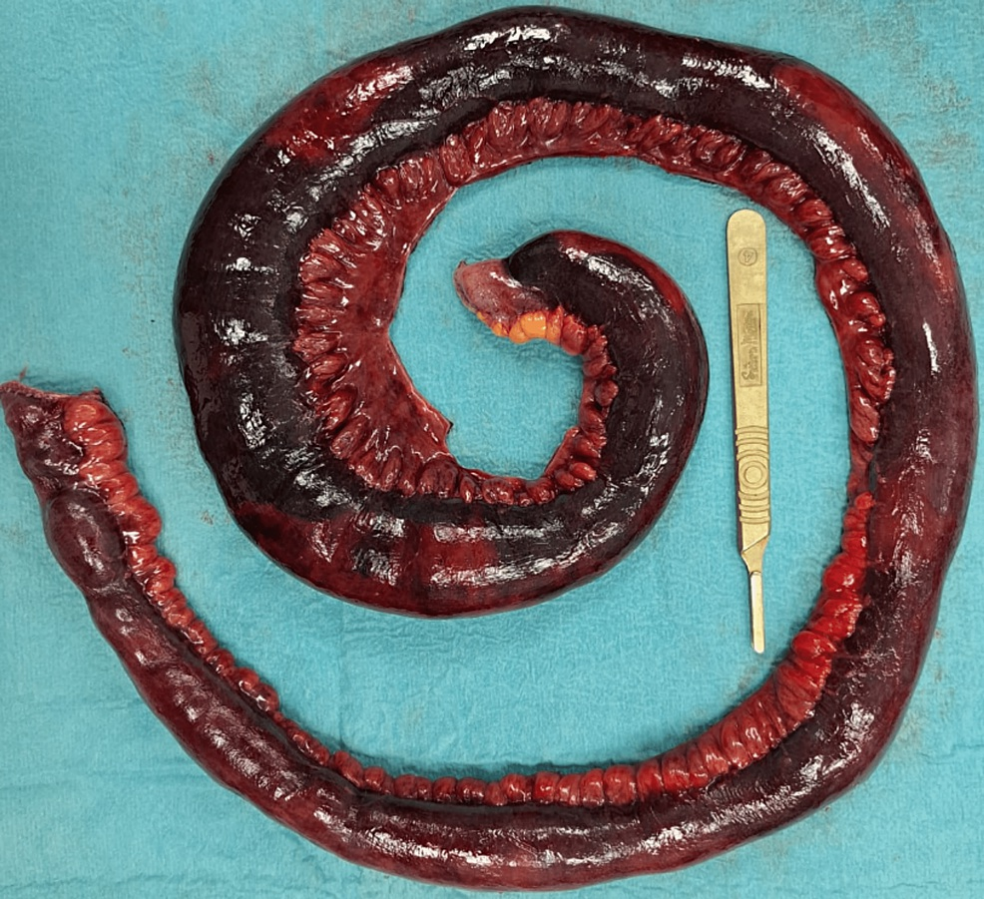
Resected segment of gangrenous intestine.

The patient was then admitted to the emergency department as a case of intestinal obstruction with intestinal ischemia and immediately posted for diagnostic laparoscopy, during which turbid hemorrhagic fluid was found intra-abdominally and the twisted, dilated gangrenous jejunal and ileal loops were identified. Laparoscopic untwisting of the affected loops was tried but failed due to a lack of intra-abdominal space. In light of these findings, the procedure was subsequently converted to an open laparotomy. A thick adhesion band from the right iliac fossa was found arising from the ileocecal junction to the mid-jejunal mesentery, leading to 360-degree anti-clockwise twisting of distal jejunal and proximal ileal loops, as evidenced in Figure 3 and Figure 4. The thickened band was attached from the right posterior peritoneal surface near the ileocecal junction to the mid-jejunum mesentery, leading to twisting of the affected loop and compromise of the blood supply to the segment. Immediately, the band was released, and the affected segment was untwisted. Hot sponge compression and 100% oxygen supply were given to facilitate blood supply to the affected intestinal loops. This maneuver improved blood supply to the distal 15 cm segment of the ileal loop; however, the remaining intestinal segment was unchanged, suggesting irreversible damage. Around 115 cm of the gangrenous, twisted affected loops were resected using a harmonic scalpel, as seen in Figure 5. Proximal jejunal loops and a healthy-looking terminal ileal segment with persistent peristalsis, up to 15 cm proximal to the ileocecal junction, were viable. A side-to-side anastomosis was performed between the proximal jejunal loop and terminal ileal segment using a linear stapler. The large bowel was inspected and found to be completely normal with good vascularity. A thorough abdominal lavage was done with warm normal saline before closing the abdomen in layers and keeping two drains in the abdominal cavity. Postoperatively, the patient recovered smoothly, started tolerating a liquid diet on the second postoperative day, and progressed to the soft diet on the fourth postoperative day. She was discharged on the sixth postoperative day and recovered well without complications.

## Discussion

The pathogenesis of adhesion formation is not entirely clear. However, the most widely accepted theory is that a disturbance of the mesothelial surface causes upregulation of the coagulation and inflammation signaling pathways [4,5]. The peritoneum and serosal surfaces are extremely sensitive, and when two or more organ surfaces are injured or irritated, it almost always results in adhesive band formation due to the healing response initiated [2,5]. The macrophages released from this response signal attract the mesothelial cells to re-epithelialize the whole surface of the disturbed bowel segment, in contrast to the conventional wound healing process. When the adhesion band develops, it is usually due to an imbalance of incomplete fibrinolysis and inadequate absorption of the fibrin breakdown [1,5].

Previous surgeries are the most common cause of the formation of adhesions, with the most common being laparotomies, as seen in our patient, and few occurrences following laparoscopy. However, it has been reported that with laparoscopic surgery, the risk of developing adhesions has decreased, with the incidence being approximately 5%. The degree of adhesion formation usually corresponds to the severity of intestinal pathology [4]. It should also be noted that adhesions can occur congenitally [3]. Gynecologic pathologies, such as endometriosis or pelvic inflammatory disease, are the most common causes of non-surgical adhesion formation [4,6]. Adhesions can also develop post-abdominopelvic radiation, which is used to treat several malignant conditions. These can be especially difficult to treat as they can be quite dense and extensive [4]. Intra-abdominal adhesive obstruction also develops due to abdominal tuberculosis; in our case, only one strong band was found, causing twisting and intestinal obstruction, which was released. The resected tissue specimen was negative for tuberculosis.

These adhesion bands pose a significant risk as they can tighten and constrict the bowel, leading to ischemia and necrosis, as seen in our patient. A preliminary diagnosis of ASBO due to adhesions is made based on the history given by the patient and the physician's clinical judgment [7]. A CT scan can reveal characteristic signs such as the "whirl sign," which is the twisting of the mesentery and omental anchoring, or the "fat-bridging sign," which is the connecting band seen in the peritoneal cavity [8]. However, only by diagnostic laparoscopy or laparotomy can the presence of adhesions be confirmed [7].

Management of ASBO can be either conservative or surgical [3,8]. Non-operative management of ASBO is preferred if there is no intestinal strangulation and is successful in 70%-90% of patients [3,9]. The use of gastrografin is one such method that can therapeutically and effectively increase intestinal motility and peristalsis. It also has advantages that lead to shorter hospital stays and recovery times; however, it does not affect mortality or morbidity [9]. It could also cause dehydration due to the effect of the fluid volume shift into the intestinal lumen; therefore, maintaining adequate fluid hydration is essential [3,9]. Surgery is necessary for those patients presenting with intestinal strangulation. Midline laparotomy with adhesiolysis is the preferred approach, although it leads to a longer hospital stay, as its benefits include increased recovery time and decreased postoperative complications, including the formation of new adhesions [4,9].

The risk of ASBO depends on the location of the adhesion, the type of surgery performed, and the various materials used during the procedure, such as sutures, gauze, and even carbon dioxide gas sufflation [3,10]. Laparoscopy is the recommended surgical approach due to the reduced risk of injury to the peritoneum and serosal surfaces, decreased contact with foreign bodies such as gauze, and the maintenance of the natural intra-abdominal climate, which reduces the risk of hypoxia [11,12]. Following the Halstedian technique, which is recommended for all operative procedures, should also be applied in these cases [9,10]. Using 2%-4% oxygen to decrease intraperitoneal hypoxia can decrease the risk of adhesion formation [3]. Using mechanical barriers such as polyethylene glycol or polylactic acid during the procedure is easy, safe, and practical. It can help separate the peritoneal serosal surfaces and allow complete, uninterrupted healing [8,10].

Szeliga and colleagues review and discuss the literature comparing the laparoscopic versus open surgical approach to adhesiolysis in the case of ASBO. They summarize that while laparoscopy is more common and recommended, realistically, it presents technical difficulties that might require converting the procedure to an open type, as seen in our case. The restricted view of the intra-abdominal cavity, especially when packed with intestines and adhesions, can be a significant obstacle when performing such diagnostic procedures [13]. They report that the conversion rate in the two studies was 39% and 42%, respectively, making this occurrence somewhat typical [13-15]. They also comment that conventional open surgery methods might prove more beneficial and effective in cases of hemodynamic instability, sepsis, or patients with a positive history of undergoing multiple surgeries and developing adhesions thereafter [13].

## Conclusions

Intestinal adhesions are a common complication of intra-abdominal surgical procedures that can lead to ASBO. It can develop due to irritation or injury to the peritoneal or serosal surfaces. While the patient's history, clinical features, and CT findings can raise suspicion for ASBO, diagnostic laparoscopy or laparotomy is preferred for confirming and treating ASBO. In those patients without intestinal strangulation, conservative management is preferred. In the presence of intestinal strangulation, operative management is recommended, more specifically a midline laparotomy with adhesiolysis. Surgeons should be mindful of the risk of adhesions developing postoperatively and should employ the recommended prevention strategies to minimize the risk. Notwithstanding these recommendations, there are some cases where the surgeon needs to employ their clinical judgment and consider conversion to open surgery.
